# The deviation of farmers’ willingness and behavior of domestic waste separation: a study on neighborhood effects and policy interventions

**DOI:** 10.3389/fpsyg.2024.1358903

**Published:** 2024-03-05

**Authors:** Xi Chen, Lirong Xing, Bowei Li, Chongcai Wang, Yue Zhang

**Affiliations:** ^1^School of Humanities and Social Sciences, Jiangsu University of Science and Technology, Zhenjiang, China; ^2^School of Economics, Shandong University of Technology, Zibo, China; ^3^College of Economics and Management, Zhejiang A&F University, Hangzhou, China; ^4^School of Management, Anhui Science and Technology University, Bengbu, China; ^5^School of Management, Hebei University, Baoding, China

**Keywords:** neighborhood effects, policy interventions, domestic waste separation, deviation of willingness and behavior, Jiangsu province

## Abstract

Based on the perspective of combining informal and formal systems, this paper empirically explores the impact of neighborhood effects and policy interventions on the deviation of farmers’ willingness and behavior of domestic waste separation (DWS) by using data from the China Land Economy Survey (CLES) and constructing a probit model. It should be explained that the neighborhood effect in this paper refers to the fact that the behavior of farmers is highly susceptible to the behavior of their neighbors in the process of production and living. The results of the study show that neighborhood effects and policy interventions have a significant negative impact on the deviation of farmers’ willingness and behavior of DWS, respectively. Comparison of marginal effects shows that neighborhood effects > environmental advocacy > reward and punishment policies. From the interaction effects as a whole, neighborhood effects and policy interventions have complementary effects on the deviation of farmers’ willingness and behavior of DWS, with the complementary effects of neighborhood effects and environmental advocacy being more significant. Heterogeneity analysis reveals that neighborhood effects completely replace the inhibitory effect of policy interventions on the deviation of high-income farmers’ willingness and behavior of DWS, but have no effect on political elite farmers. The interaction between neighborhood effects and policy interventions has complementary effects on low-income farmers and ordinary farmers, with the complementary effects of neighborhood effects and environmental advocacy being more significant.

## 1 Introduction

Currently, the world is facing a war on the waste crisis. The United Nations Environment Programme (UNEP) stated at the Global Partnership on Waste Management that “the problem of waste is evolving into a crisis with global dimensions” (CCTV, 2012). According to World Bank statistics, the amount of waste produced in East Asia and the Pacific is 468 million tons per year (World Bank, 2022). If no action is taken, the amount of global waste will increase by 70% to as much as 3.4 billion tons by 2050 (World Bank, 2018). Especially after global outbreaks of infectious diseases, the need for public health governance has received increasing human attention (Chen et al., 2023a). China is one of those countries. China’s rural domestic waste production is increasing dramatically at a rate of 8 ~ 10% per year (Zhang et al., 2019). This has led to the challenge of “garbage-encircled villages” in some rural areas of China (Chen et al., 2023b). International management experience tells us that source separation is a viable program for waste management (Paglietti et al., 2016; Manomaivibool et al., 2018). Unfortunately, source separation of domestic waste in China’s rural areas has only just begun and is in a small-scale pilot phase (Chen et al., 2023a). Then, how can rural domestic waste management be effectively achieved?

From the perspective of governance models, Chinese government advocates public-private partnership (PPP) project, which involves a number of entities. But the most prominent problem faced by this project is the low level of farmer participation (Chen et al., 2023a). From the perspective of the main body of governance, farmers are not only the main participants but also the direct beneficiaries. Therefore, farmers’ participatory attitudes are crucial for rural domestic waste management. According to the CLES conducted by Nanjing Agricultural University, in Jiangsu Province, farmers’ willingness to participate in household waste separation is high (89.94%), but their practical behavior is low (53.06%). Surveys by related scholars have found similar results (Jia and Zhao, 2020; Zhou et al., 2022). It can be seen that reducing the deviation of farmers’ willingness and behavior of DWS is crucial for improving the rural habitat. Then the problem is, how can we reduce it?

At present, domestic waste management research is gradually being focused on by many scholars. In terms of research regions, previous scholars have looked more on urban domestic waste (Wang et al., 2019; Jin et al., 2021; Sun and Asari, 2023), neglecting the examination of rural residents’ domestic waste separation (Xu et al., 2017; Knickmeyer, 2020; Zhou et al., 2022). In fact, in terms of urban–rural, solid waste has grown faster in rural areas than in county areas (Chen et al., 2023c). In the limited study on waste separation for rural residents, the influencing factors are mainly categorized as internal and external factors (Chen et al., 2023a,b). Internal factors mainly include environmental concern (Stern et al., 1995), pollution perception (Jia et al., 2021), perceived environmental regulation (Xu et al., 2023) and so on. External factors mainly include economic subsidy (Zen and Siwar, 2015; Xu et al., 2018), environmental monitoring (Li X. et al., 2019), fiscal decentralization (Ma et al., 2021), digital governance (Chen et al., 2023a) and so on. All of these will have an impact on farmers’ waste separation behavior.

The possible marginal contributions of this paper are as follows. First, exploring the impact of neighborhood effects on the deviation of farmers’ willingness and behavior of DWS. In fact, stemming from geo-cultural and collectivist ideas, Chinese residents are more susceptible to the views or behaviors of others (Eun et al., 2015). Therefore, in the special socio-cultural context of China, it would be biased to ignore the social attributes of farmers and analyze their behavioral decisions in isolation from the social space in which they are located (Shi et al., 2022). Neighborhood effects are widespread in rural China and are an informal system with strong social attributes. Currently, few scholars explore the problem based on neighborhood effects perspective. Second, based on the perspective of combining informal and formal systems, neighborhood effects and policy interventions are incorporated into the same analytical framework to explore the impact of their interactive effects on the deviation of farmers’ willingness and behavior of DWS. Few scholars have explored the impact of the interactive effects of neighborhood effects and policy interventions on the deviation of farmers’ willingness and behavior of DWS.

The rest of this paper is organized as follows. The second part is the theoretical analysis. The third part includes data sources, indicators and methods. The fourth part is the analysis of the empirical results. The fifth part is the main research conclusions and policy recommendations.

## 2 Theoretical hypothesis

According to social interaction theory, neighborhood effects can impact on the deviation of farmers’ willingness and behavior of DWS through two pathways: endogenous and situational interactions (Durlauf and Ioannides, 2010). Endogenous interactions are at the root of the social multipliers unleashed by neighborhood effects, emphasizing the herd effect of behavioral outcomes. Situational interaction emphasizes the demonstrative effect of behavioral outcomes. The specific analyses are as follows.

First, neighborhood effects reduce the deviation of farmers’ willingness and behavior of DWS by influencing the psychological burden, emotional support and information sharing of farmers, specifically reflecting the herd effect in endogenous interactions. From the perspective of psychological burden, in a relational society, the social attribute of neighborhood effects has a normative constraint function, which can reduce the deviation of farmers’ willingness and behavior of DWS. Because farmers care about their neighbors’ opinion of them in order to maintain their reputation (Li and Su, 2007; Oetzel et al., 2008). If the behavior of littering domestic waste is resisted by the neighborhood, the continuation of the behavior will be subject to public opinion pressure from the neighborhood and increase the psychological burden, which is detrimental to their reputation. From an emotional support perspective, a sense of belonging to the village will motivate more non-direct stakeholders to participate in collective action (Klandermans, 2002). The social attributes of the neighborhood effects can enhance interaction and communication among residents and promote their sense of belonging and identity with the village. Psychological studies show that farmers with a sense of belonging care more about the collective good (Lu, 2008). The human geography perspective suggests that place attachment and place identity are important ways of embodying of a sense of belonging to a place, which can motivate people to display supportive attitudes and pro-environmental behaviors towards environmental protection (Vaske and Kobrin, 2001; Kyle et al., 2003). From the perspective of information sharing, the exchange of information between farmers about DWS not only reduces the cost of information searching, but also enjoys the pleasure of a common topic in the process of communication, which enhances the self-efficacy of farmers to recycle (Liu et al., 2020). It can be seen that neighborhood effects can impact on farmers’ willingness and behavior of DWS at the village scale.

Second, neighborhood effects reduce the deviation of farmers’ willingness and behavior of DWS by observational learning and breaking down information barriers, specifically embodied in the demonstration effect in situational interactions. Classical economists believed that people behave in accordance with the principle that marginal revenue and marginal cost are equal (Li et al., 2022). Indeed, farmers are not only limitedly rational but also exhibit risk aversion to future uncertainty in their decision-making process (Scott, 1977). Therefore, farmers will make decisions based on the principle of maximizing benefits. In particular, *a priori* information about cost–benefit is an important guide to farmers’ DWS decisions. In the real world, farmers can obtain information on the costs (time costs and economic costs) and benefits (economic benefits and social benefits) of DWS from other farmers by observation and learning and information exchange. They use this as a reference to adjust their own decisions. If the *a priori* information obtained through information exchange with other farmers judges that the expected returns from recycling behavior are relatively substantial, then farmers will follow suit. For those who follow suit, the farmers who are the first to implement recycling behavior act as guides and demonstrations. Therefore, this paper proposes the following hypothesis.

*H1*: Neighborhood effects have a significant negative impact on the deviation of farmers’ willingness and behavior of DWS.

From the perspective of economics, there are obvious negative externalities associated with the discharge of domestic waste by rural residents, which can easily lead to moral hazard and free-riding behavior (Chen et al., 2023a). Therefore, it is necessary for governments to adopt certain incentive or constraint policies to internalize the externalities of environmental pollution (Li F. N. et al., 2019). Currently, policy interventions on DWS in rural areas of China are mainly reward and punishment policies (economic subsidies and penalty policies) and environmental advocacy. According to reinforcement theory, economic subsidies are positive reinforcement measures and penalties are negative reinforcement measures. A number of studies confirm that farmers act as economic agents and that economic subsidies can increase private marginal benefits and have an incentive effect on DWS (Owusu et al., 2013; Boonrod et al., 2015; Xu et al., 2018). Penalty policies have regulatory constraints and warning effects that can also incentivize pro-environmental behavior among farmers (Loan et al., 2017; Hu et al., 2022). From the perspective of environmental advocacy, information advocacy has a direct positive impact on residents’ willingness to separate waste (Wang et al., 2019; Zhang et al., 2019). In addition, environmental education can promote rural residents’ participation in domestic waste management by increasing their awareness of environmental issues as well as their level of concern (Kil et al., 2014; Loan et al., 2017; Li F. N. et al., 2019; Li X. et al., 2019). Therefore, this paper proposes the following hypothesis.

*H2*: Policy interventions have a significant negative effect on the deviation of farmers’ willingness and behavior of DWS.

Neighborhood effects and policy interventions are two different governance instruments that have different ways and processes of influencing farmers’ willingness and behavior of DWS. So, is there a complementary or substitution effects between the two? Based on the governance attributes, neighborhood effects can exert informal institutional constraints at the level of ideology to increase farmers’ perception of waste separation and promote pro-environmental behavior. Policy interventions can promote farmers’ participation in DWS by increasing their marginal benefits, default costs, and raising their perception of waste separation (Owusu et al., 2013; Kil et al., 2014; Boonrod et al., 2015; Loan et al., 2017; Xu et al., 2018; Wang et al., 2019). In addition, it has been found that due to the neighborhood effects, the impacts of exogenous policies are no longer confined to specific individuals, but rather “reverberate” among certain groups, generating a social multiplier effect that amplifies the effects of public policies (Becker, 1974; Becker and Murphy, 2000). Therefore, this paper proposes the following hypothesis.

*H3*: Neighborhood effects and policy interventions have complementary effects on reducing the deviation of farmers’ willingness and behavior of DWS.

**Figure 1:**
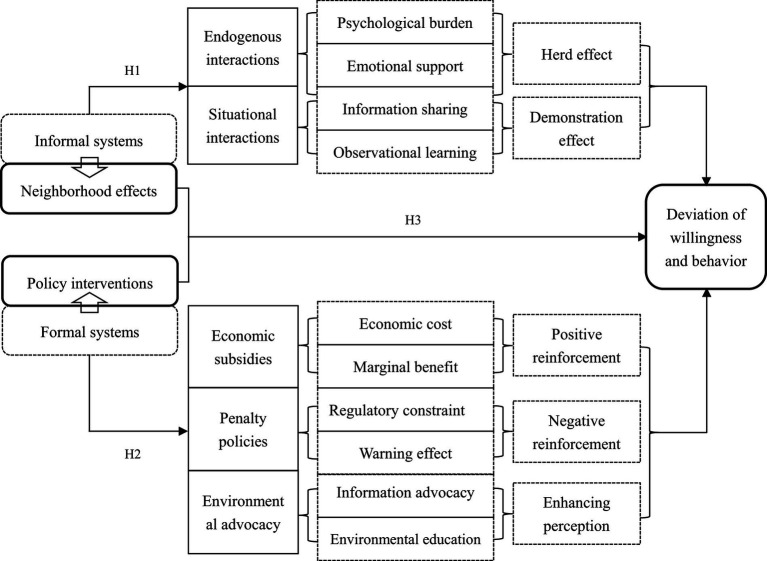
The impact mechanism of neighborhood effects and policy interventions on the deviation of farmers’ willingness and behavior of DWS.

Based on the above analysis, this study mapped the mechanisms of the impact of neighborhood effects and policy interventions on the deviation of farmers’ willingness and behavior of DWS (Figure 1).

## 3 Methodologies

### 3.1 Data sources

Data from the China Land Economy Survey (CLES) conducted by Nanjing Agricultural University in 2021. The survey covered rural areas in Jiangsu Province. The research questionnaire consists of a questionnaire for rural residents and a questionnaire for villages. The rural resident questionnaire covers land use, family assets, rural governance, etc. The village questionnaire covers demographic characteristics, land situation, collective economy and external environment. The research activity uses PPS sampling, the total sample of 2,627 households. After removing the invalid samples from the questionnaire, the final sample size was 2,342.

### 3.2 Variables

(1) Dependent variable. The dependent variable in this paper is the deviation of farmers’ willingness and behavior of domestic waste separation (*DWB*). Previous scholars have paid more attention to farmers’ willingness to separate domestic waste (Han et al., 2019; Chen et al., 2023b) or separation behavior (Jia et al., 2021; Chen et al., 2023a). However, few scholars have examined whether there is a deviation between farmers’ willingness and behavior of domestic waste separation (DWS). Deviation of willingness and behavior in this paper only refers to the “willing without behavior” farmers. In addition, the indicators measure. In this paper, judgements are made by the following two questions. Are you willing to separate domestic waste (0 = No; 1 = Yes)? Do you carry out domestic waste separation (0 = No; 1 = Yes)? Based on the two questions above, if there is willingness and behavior, the value is 0, which means that the willingness is consistent with the behavior.

(2) Core independent variable. The core independent variables in this paper are the neighborhood effects (*NEE*) and the policy interventions (*POI*), respectively. First, neighborhood effects (*NEE*). Currently, there are three main approaches used to measure it.①A multi-dimensional indicator system is constructed to measure. The indicator system is designed from three aspects: observation and learning, experience sharing and social norms, and then the neighborhood effects are measured by using factor analysis method (Tang and Luo, 2022). ② A single indicator is measured. In addition to individual farmers, the mean value of a particular behavior of the surveyed farmers in the village is considered as a neighborhood effects (Manski, 1993; Lee, 2007; Zuo, 2020). ③ Natural spatial distances are used for measurement. Some researchers define a household’s neighborhood as a geographical area that can be reached by a 5-min walk or within a radius of 50 and 100 m (Hur et al., 2010; Hong and He, 2020). Given the availability of survey data, this paper decided to use a single indicator to measure neighborhood effects. It is measured by the mean value of the DWS behavior of the other farmers surveyed in the village, excluding the farmer himself.

Second, policy interventions (*POI*). This paper focuses on reward and punishment policies (*RPP*) and environmental advocacy (*ENA*). Reward and penalty policy (*RPP*), which mainly consists of economic incentives and penalties, is an important tool as a means to enhance farmers’ DWS behavior (Viscusi et al., 2011; Owusu et al., 2013; Xu et al., 2018). Environmental advocacy (*ENA*), as an external stimulus, also impacts farmers’ willingness and behavior of DWS (Han et al., 2019; Li F. N. et al., 2019; Li X. et al., 2019; Xu et al., 2023). The measurement of these two indicators is mainly discerned by the following two questions in the questionnaire. Regarding the domestic waste separation in rural areas, has the government implemented rewards and penalties (0 = No; 1 = Yes)? Has the government publicized the separation of rural domestic waste (0 = No; 1 = Yes)?

(3) Control variables. In order to reduce omitted variable bias, and taking into account the experience of existing studies (Xu et al., 2023; Chen et al., 2023a,b), this study controls for the individual characteristics, household characteristics and village characteristics of the respondents, respectively.

Individual and household characteristics. ① Sex of the respondent (*SEX*). ② Age of the respondent (*AGE*), expressed as the actual age of the respondent. ③ Respondent’s educational attainment (*EDU*), expressed as the respondent’s actual years of schooling. ④ Respondents’ pollution perceptions (*POP*). Farmers’ level of perception of domestic waste pollution is also an important factor influencing their sorting willingness and behavior (Jia et al., 2021). The questionnaire is “What do you think is the impact of dumping and non-separation of domestic waste on the rural ecological environment (deterioration of water quality, contamination of soil, etc.)?.” ⑤ Family Resident Population (*FRP*). In general, the larger the household, the more domestic waste is generated, which has an impact on farmers’ domestic waste management behavior. The questionnaire is “How many people in your household are permanent residents (living in your household 6 months or more of the year)?.” ⑥ Family Political Status (*FPS*). The questionnaire is “Is your family a party member household?”

Village characteristics. ① Public Waste Sanitation Facility (*PWSF*). The questionnaire is “Number of rubbish bins/litter bins in the village.” ② Social supervision (*SOCS*). Popular participation in social supervision has different levels of influence on farmers’ domestic waste management behavior (Govindan et al., 2022; Sun et al., 2022). The questionnaire is “What measures does the village take to monitor littering and dumping?.” If the farmer chooses to have no supervision measures, the value is 0, otherwise, the value is 1. ③ Number of enterprises (*NUE*). This indicator provides some indication of the level of economic development of the village and is expressed in terms of the number of enterprises that have invested in the village in 2021. ④ PPP projects (*PPP*). PPP projects are actually public-private partnerships (PPP), a market-based exploration by the Chinese government to carry out rural habitat improvement. The questionnaire is “Is there any rural waste management PPP project in the village?.” Table 1 shows the description and statistics of the relevant variables.

**Table 1:** Variable descriptive statistics.

Variable	Definition	Mean	Std. dev.
*DWB*	0 = Willingness and behavior; 1 = Willingness without behavior	0.4133	0.4925
*NEE*	Mean value of DWS behavior of other interviewed farmers in the same village	0.5290	0.2049
*RPP*	0 = No,1 = Yes	0.2685	0.4433
*ENA*	0 = No,1 = Yes	0.8509	0.3562
*SEX*	0 = female,1 = male	0.9245	0.2643
*AGE*	Actual age of respondents (year)	63.3318	10.6200
*EDU*	Respondents’ actual years of schooling (year)	7.3100	3.6527
*POP*	5-level Likert scale, 1 = very small, 5 = very large	4.1594	0.8063
*FRP*	Number of actual permanent residents in the household (unit)	3.0389	1.5842
*FPS*	0 = No,1 = Yes	0.3101	0.4626
*PWSF*	Number of village dustbins (unit)	518.0496	875.9716
*SOCS*	0 = No,1 = Yes	0.8696	0.3368
*NUE*	Number of village enterprises (unit)	1.1511	2.7918
*PPP*	0 = No,1 = Yes	0.2847	0.4514

### 3.3 Methods

Considering that the deviation of farmers’ willingness and behavior of DWS is a 0–1 variable. Therefore, we adopt the probit model to explore it. The model form is as follows:


(1) $$DWB=\alpha_{0}+\alpha_{1}NEE+\alpha_{2}POI+\alpha_{3}Z+\varepsilon$$

In order to verify the substitution or complementary effects of neighborhood effects and policy interventions, this paper constructs equation (2) by adding the interaction term between the two into the model.


(2) $$DWB=\alpha_{0}+\alpha_{1}NEE+\alpha_{2}POI+\alpha_{3}NEE\times POI+\alpha_{4}Z+\varepsilon$$

In Equations (1, 2), *DWB* is the deviation of farmers’ willingness and behavior of DWS. *NEE* is the neighborhood effects. *POI* is the policy intervention, which include reward and punishment policies (*RPP*) and environmental advocacy (*ENA*). *Z* is a control variable, which mainly consists of individual characteristics, household characteristics and village characteristics. α is the coefficient of the independent variable. ε is a random error term.

## 4 Empirical results analysis

### 4.1 The estimated results based on the total sample

First, correlation test. The results show that the variance inflation factor (VIF) of each explanatory variables is less than 1.74 indicating that the regression model does not suffer from multicollinearity. Second, to reduce estimation bias, all regressions in this paper are estimated by using robust standard errors for clustering at the village level and the inclusion of control variables. Table 2 reports the average marginal effect results.

**Table 2:** The estimated results based on the total sample.

Variable	M1	M2	M3	M4	M5
	Marginal effects	Marginal effects	Marginal effects	Marginal effects	Marginal effects
*NEE*	−0.7260*** (0.0697)				−0.6480*** (0.0815)
*RPP*		−0.2620*** (0.0305)		−0.2210*** (0.0314)	−0.2170*** (0.0254)
*ENA*			−0.3200*** (0.0337)	−0.2660*** (0.0329)	−0.2350*** (0.0316)
*SEX*	0.0034 (0.0377)	−0.0048 (0.0378)	−0.0147 (0.0375)	−0.0123 (0.0379)	0.0008 (0.0389)
*AGE*	0.0045*** (0.0015)	0.0046*** (0.0013)	0.0050*** (0.0014)	0.0045*** (0.0013)	0.0037*** (0.0013)
*EDU*	0.0024 (0.0036)	0.0022 (0.0037)	0.0032 (0.0040)	0.0031 (0.0039)	0.0032 (0.0037)
*POP*	−0.0408** (0.0165)	−0.0378** (0.0164)	−0.0404** (0.0167)	−0.0318** (0.0157)	−0.0250* (0.0141)
*FRP*	−0.0139** (0.0059)	−0.0184*** (0.0066)	−0.0161** (0.0067)	−0.0140** (0.0065)	−0.0073 (0.0056)
*FPS*	−0.0691** (0.0306)	−0.0417 (0.0305)	−0.0644** (0.0292)	−0.0393 (0.0280)	−0.0389 (0.0272)
*PWSF*	−6.81e-06 (1.42e-05)	−7.30e-05*** (2.61e-05)	−8.00e-05*** (2.92e-05)	−6.59e-05** (2.59e-05)	6.17e-06 (1.44e-05)
*SOCS*	−0.0156 (0.0159)	−0.0715** (0.0353)	−0.0766* (0.0404)	−0.0812** (0.0344)	−0.0388** (0.0172)
*NUE*	−0.0084 (0.0054)	−0.0200** (0.0083)	−0.0283*** (0.0095)	−0.0200** (0.0083)	−0.0008 (0.0043)
*PPP*	−0.0200 (0.0222)	−0.0485 (0.0627)	−0.0320 (0.0567)	−0.0313 (0.0579)	0.0004 (0.0219)
Log likelihood	−954.6542	−941.7950	−952.3240	−913.1319	−880.0847
Wald chi^2^	292.71	183.50	150.51	217.64	487.79
Prob > chi^2^	0.0000	0.0000	0.0000	0.0000	0.0000
Observations	1,557	1,543	1,556	1,543	1,543

***, **, * Represent significant at 1, 5, 10% confidence level, respectively.

M1 in Table 2 show that neighborhood effects (*NEE*) have a significant negative effect (at 1% level) on the deviation of farmers’ willingness and behavior of DWS. This indicates that there is a neighborhood effects on the deviation of farmers’ willingness and behavior of DWS. Specifically, neighborhood effects contribute 72.60% to reducing the deviation of farmers’ willingness and behavior of DWS, and its effect cannot be ignored. In other words, in villages, when neighbors around them carry out DWS, farmers within other villages will also actively participate in DWS. This is in keeping with a Chinese proverb: “What’s near cinnabar goes red, and what’s next to ink turns black.” Therefore, H1 is verified.

M2 and M3 in Table 2 show reward and penalty policy (*RPP*) and environmental advocacy (*ENA*) have a significant negative effect (at 1% level) on the deviation of farmers’ willingness and behavior of DWS, respectively. M4 in Table 2 is the combined effect of policy interventions. Specifically, *RPP* and *ENA* contribute 22.10 and 26.60%, respectively, to reducing the deviation of farmers’ willingness and behavior of DWS. In terms of contribution, *ENA* is slightly more effective than *RPP*. It can be seen that policy interventions, as an external stimulus, have strong positive externalities. On the one hand, incentives and constraints can be used to increase the transformation of farmers’ willingness to separate domestic waste into behavior. On the other hand, *ENA* can be used to raise the pollution perception and policy recognition of domestic waste, thus facilitating the transformation of their willingness to separate domestic waste into behavior. H2 is verified.

M5 in Table 2 show that neighborhood effects (*NEE*), reward and punishment policy (*RPP*) and environmental advocacy (*ENA*) still have a significant negative effect (at 1% level) on the deviation of farmers’ willingness and behavior of DWS under the combined impacts of neighborhood effects and policy interventions. Specifically, the marginal effects of *NEE*, *RPP*, and *ENA* are 0.6480, 0.2170, and 0.2350, respectively, which shows that *NEE* is superior. The village has long been the basic unit of grassroots governance in China. In village governance, farmers’ production and livelihood behavior is highly susceptible to neighborhood effects (Manski, 2000; Eun et al., 2015). Disposal of DWS by farmers is strongly individually subjective and susceptible to past path dependency. *RPP* and *ENA*, while having some incentive effects, have limited sustainability of policy effects (Schultz et al., 1995) and do not provide sufficient incentives for everyone (Sun and Asari, 2023). Therefore, in the current governance of rural habitats, neighborhood effects remain an important force to inhibit the deviation of farmers’ willingness and behavior of DWS.

Among the control variables, *AGE* has a significant positive effect on the deviation of farmers’ willingness and behavior of DWS. It can be seen that the older the farmer is, the less conducive it is to the transformation of willingness to separate domestic waste into behavior. *POP* has a significant negative effect on the deviation of farmers’ willingness and behavior of DWS. This indicates that the higher the level of farmers’ perception of domestic waste pollution is the more conducive to achieving the transformation of farmers’ willingness to separate into behavior. It is consistent with the findings of related scholars (Jia et al., 2021; Xu et al., 2023). *SOCS* has a significant negative effect on the deviation of farmers’ willingness and behavior of DWS. It can be seen that social supervision can improve the conversion of farmers’ willingness to separate domestic waste into behavior.

### 4.2 Analysis of the interactive effects of neighborhood effects and policy interventions

In this section, an interaction term between neighborhood effects and policy interventions was constructed to test the complementary or substitution effects of neighborhood effects and policy interventions in reducing the deviation of farmers’ willingness and behavior of domestic waste separation (DWS). Specific test results are detailed in Table 3.

**Table 3:** The regression results of the interactive effects of neighborhood effects and policy interventions.

Variable	M1	M2	M3	M4
	Coef.	Marginal effects	Coef.	Marginal effects
*NEE*	−2.1050*** (0.3320)	−0.7010*** (0.1030)	−1.0690** (0.4450)	−0.3590** (0.1500)
*RPP*	−0.7130*** (0.2330)	−0.2380*** (0.0756)		
*ENA*			−0.3980* (0.2330)	−0.1340* (0.0787)
*NEE×RPP*	−0.0953 (0.447)	−0.0317 (0.1490)		
*NEE×ENA*			−0.9590** (0.4840)	−0.3220** (0.1610)
*Controlled variables*	YES	YES	YES	YES
Log likelihood	−903.4005	−903.4005	−919.5005	−919.5005
Wald chi^2^	536.98	536.98	428.04	428.04
Prob > chi^2^	0.0000	0.0000	0.0000	0.0000
Observations	1,543	1,543	1,556	1,556

***, **, * represent significant at 1, 5, 10% confidence level, respectively.

M1 and M2 in Table 3 show that the interaction term between neighborhood effects (*NEE*) and reward and punishment policies (*RPP*) is negative but not significant. This indicates that the complementary effect between the two is not significant. M3 and M4 in Table 3 show that the interaction term between *NEE* and environmental advocacy (*ENA*) is significantly negative at the 5% level. This indicates that there is a complementary effect between the two. In an acquaintance society, neighborhood communication is the traditional way of communication in rural China. However, the sustainability of the effects of the implementation of *RPP* is limited (Schultz et al., 1995) and does not provide sufficient incentives for everyone (Sun and Asari, 2023). These may be important reasons why *NEE* and *RPP* have not yet developed a complementary effect.

In addition, environmental advocacy (*ENA*) has a strong guiding role (Han et al., 2019). In terms of effect, *ENA* not only enhances farmers’ perception of waste separation from within the psyche, but also expands the breadth and depth of publicity on *RPP* to achieve system recognition. In terms of communication methods, compared to *RPP*, *ENA* can not only unilaterally deliver environmental information to farmers, but also face-to-face in both directions. Overall, the interaction term between neighborhood effects (*NEE*) and policy intervention (*POI*) is negative. H3 is verified. Therefore, in order to reduce the deviation of farmers’ willingness and behavior of domestic waste separation (DWS), the relevant government departments should not only pay attention to the informal system of *NEE*, but also expand the formal institutional constraints of *ENA* and *RPP*, so as to bring into play the synergistic effect of the two.

### 4.3 Robustness tests

(1) Replacement model test. To further test the reliability of the model estimation results, this paper adopted Logit model to regress again. M1 to M5 in Table 4 show that neighborhood effects and policy interventions have a significant negative effect on the deviation of farmers’ willingness and behavior of domestic waste separation (DWS), which is consistent with the results estimated in Table 2 (from M1 to M5). M6 and M7 in Table 4 show that the interaction effects of neighborhood effects and policy interventions are almost identical to the results estimated in Table 3 (M2 and M4). Therefore, the estimation results are robust in this paper.

**Table 4:** The regression results of the replacement model test.

Variable	Replacement model						
	M1	M2	M3	M4	M5	M6	M7
*NEE*	−0.7240*** (0.0714)				−0.6470*** (0.0821)	−0.6890*** (0.1020)	−0.3640** (0.1500)
*RPP*		−0.2660*** (0.0313)		−0.2220*** (0.0322)	−0.2190*** (0.0260)	−0.2060*** (0.0764)	
*ENA*			−0.3220*** (0.0347)	−0.2670*** (0.0337)	−0.2350*** (0.0326)		−0.1370* (0.0809)
*NEE×RPP*						−0.1020 (0.1570)	
*NEE × ENA*							−0.3150* (0.1630)
*Controlled variables*	Yes	Yes	Yes	Yes	Yes	Yes	Yes
Log likelihood	−954.9618	−942.2584	−952.4196	−913.4385	−880.5061	−903.6906	−919.7906
Wald chi^2^	229.38	152.96	133.29	186.21	375.57	423.52	361.75
Prob > chi^2^	0.0000	0.0000	0.0000	0.0000	0.0000	0.0000	0.0000
Observations	1,557	1,543	1,556	1,543	1,543	1,543	1,556

***, **, * Represent significant at 1, 5, 10% confidence level, respectively.

(2) Winsorized test. In order to prevent the influence of extreme values on the results, this paper performs the Winsorized test (at the 1% level) for continuous variables (*NEE*, *FRP* and *PWSF*). M1 to M5 in Table 5 show that neighborhood effects and policy interventions have a significant negative effect on the deviation of farmers’ willingness and behavior of DWS, which is consistent with the results estimated in Table 2 (from M1 to M5). M6 and M7 in Table 5 show that the interaction effects of neighborhood effects and policy interventions are almost identical to the results estimated in Table 3 (M2 and M4). Therefore, the estimation results are robust in this paper.

**Table 5:** The regression results of the Winsorized test.

Variable	Winsorized test						
	M1	M2	M3	M4	M5	M6	M7
*NEE*	−0.7260*** (0.0698)				−0.6510*** (0.0813)	−0.7000*** (0.1030)	−0.3590** (0.1500)
*RPP*		−0.2620*** (0.0310)		−0.2200*** (0.0319)	−0.2180*** (0.0258)	−0.2290*** (0.0776)	
*ENA*			−0.3190*** (0.0337)	−0.2650*** (0.0330)	−0.2340*** (0.0317)		−0.1330* (0.0786)
*NEE × RPP*						−0.0507 (0.1550)	
*NEE × ENA*							−0.3230** (0.1610)
*Controlled variables*	Yes	Yes	Yes	Yes	Yes	Yes	Yes
Log likelihood	−944.3545	−931.3774	−941.8713	−903.0987	−870.3003	−893.3193	−909.4903
Wald chi^2^	289.59	187.36	148.11	216.62	475.55	538.98	433.09
Prob > chi^2^	0.0000	0.0000	0.0000	0.0000	0.0000	0.0000	0.0000
Observations	1,537	1,523	1,536	1,523	1,523	1,523	1,536

***, **, * Represent significant at 1, 5, 10% confidence level, respectively.

### 4.4 Analysis of the heterogeneity of effects

In order to examine the heterogeneity of neighborhood effects, policy interventions and the interaction of the two on the deviation of farmers’ willingness and behavior of domestic waste separation (DWS), this study will further explore based on differences in household structure. According to family capital theory, family capital is a special type of social capital that exists only within family relationships and is a key strategic resource that impacts individual decisions (Hoffman et al., 2006). This study divides the family structure into elite and ordinary families. Elite families are divided into economic and political elites based on their economic income and political status. The specific research is described below.

(1) Based on the analysis of economic income differences. Referring to related studies (Li et al., 2022), this paper is based on villages, which are classified into high-income farmers (economic elites) and low-income farmers based on mean value of the total household income. M1 to M4 in Table 6 show that neighborhood effects (*NEE*) and Policy interventions are significantly negative at least at the 10% level for both high-income and low-income farmers (except for M4). From the interaction terms, the marginal effects for high-income farmers are all positive but not significant. The marginal effects are all negative for low-income farmers, but only the interaction term between *NEE* and *ENA* is significant at the 1% level.

**Table 6:** Estimation results for the heterogeneity of effects based on income.

Variable	High-income farmers		Low-income farmers	
	M1	M2	M3	M4
*NEE*	−0.6570*** (0.1510)	−1.2680** (0.5560)	−0.6910*** (0.1180)	−0.1720 (0.1840)
*RPP*	−0.2690* (0.1540)		−0.2200*** (0.0713)	
*ENA*		−0.5500* (0.3190)		−0.0730 (0.0877)
*NEE × RPP*	0.0099 (0.2820)		−0.0561 (0.1500)	
*NEE × ENA*		0.6750 (0.6360)		−0.4940*** (0.1830)
*Controlled variables*	Yes	Yes	Yes	Yes
Log likelihood	−250.6711	−258.9677	−650.6097	−654.9159
Wald chi^2^	77.71	123.09	631.06	349.55
Prob > chi^2^	0.0000	0.0000	0.0000	0.0000
Observations	409	414	1,134	1,142

***, **, * Represent significant at 1, 5, 10% confidence level, respectively.

Taking the above descriptive analyses together, the following findings can be made. First, overall, neighborhood effects and policy interventions have a negative impact on both high-income and low-income farmers. Second, whether or not neighborhood effects are involved, policy interventions do not have a significant impact on high-income farmers, but have a dampening effect on low-income farmers. In other words, for high-income farmers, neighborhood effects completely replace the disincentive effect of policy intervention. For low-income farmers, there are complementary effects of neighborhood effects and policy interventions, especially environmental advocacy. It can be seen that local governments need to design different ways of environmental advocacy and reward and punishment measures for farmers with different incomes in order to improve the effectiveness of policy interventions in future rural household waste separation governance.

(2) Based on the analysis of political status differences. Referring to related research (Wu, 2010), incumbent village cadres, villagers’ representatives or villagers’ party members are included in the category of rural elites. Compared to ordinary farmers, these farmers are known as political elites because of their political status. M1 to M4 in Table 7 show that neighborhood effects are significantly negative at least at the 5% level for both politically elite and ordinary farmers (except for M4). From the interaction terms, none of the marginal effects for political elite farmers are significant. The marginal effects for ordinary farmers are all negative, but only the interaction term between *NEE* and *ENA* is significant at the 5% level.

**Table 7:** Estimation results for the heterogeneity of effects based on political status.

Variable	Politically elite farmers		Ordinary farmers	
	M1	M2	M3	M4
*NEE*	−0.6280*** (0.1330)	−0.9930** (0.3890)	−0.7550*** (0.1460)	−0.2270 (0.1970)
*RPP*	−0.2480** (0.1160)		−0.2230** (0.1040)	
*ENA*		−0.3630** (0.1630)		−0.0829 (0.1070)
*NEE × RPP*	−0.0295 (0.2310)		−0.0417 (0.2010)	
*NEE × ENA*		0.4260 (0.3670)		−0.5240** (0.2150)
*Controlled variables*	YES	YES	YES	YES
Log likelihood	−317.1531	−336.8977	−583.9822	−577.3487
Wald chi^2^	170.29	147.56	452.14	172.73
Prob > chi^2^	0.0000	0.0000	0.0000	0.0000
Observations	573	578	970	978

***, **, * Represent significant at 1, 5, 10% confidence level, respectively.

Taking the above descriptive analyses together, the following findings can be made. First, overall, neighborhood effects and policy interventions have a negative impact on both politically elite and ordinary farmers. Second, the interaction between neighborhood effects and policy interventions has no effect on political elite farmers, while there are complementary effects on ordinary farmers, especially in environmental advocacy. It can be seen that local governments should pay special attention to stimulating the role model of politically elite farmers in the future rural domestic waste separation governance.

## 5 Conclusion and implications

In rural domestic waste management, farmers are not only the main participants but also the direct beneficiaries. Therefore, farmers’ attitudes towards participation are closely related to the quality of rural domestic waste management. However, farmers face the dilemma of high willingness and low behavior in domestic waste separation (DWS). Then, how can we effectively reduce the deviation of farmers’ willingness and behavior of DWS? Therefore, based on the perspective of combining informal and formal systems, this paper empirically explores the impact of neighborhood effects and policy interventions on the deviation of farmers’ willingness and behavior of DWS by using data from the CLES and constructing a probit model.

The results of the study show that neighborhood effects and policy interventions have a significant negative impact on the deviation of farmers’ willingness and behavior of DWS, respectively, and the findings still hold after robustness tests. Comparison of marginal effects shows that neighborhood effects > environmental advocacy > reward and punishment policies. From the interaction effects as a whole, neighborhood effects and policy interventions have complementary effects on the deviation of farmers’ willingness and behavior of DWS, with the complementary effects of neighborhood effects and environmental advocacy being more significant. Heterogeneity analysis reveals that neighborhood effects completely replace the inhibitory effect of policy interventions on the deviation of high-income farmers’ willingness and behavior of DWS, but have no effect on political elite farmers. The interaction between neighborhood effects and policy interventions has complementary effects on low-income farmers and ordinary farmers, with the complementary effects of neighborhood effects and environmental advocacy being more significant. Accordingly, the paper makes the following recommendations:

(1) Establishing a mechanism for positive interaction between neighbors. Rural areas in China are typical societies of acquaintances. Policy interventions suffer from problems such as persistence. Therefore, rural waste separation governance still needs to give full play to neighborhood effects. On the one hand, it is necessary to give full play to the demonstration and leading role of elite farmers within the village. On the other hand, it is necessary to actively cultivate a good atmosphere for neighborhood interaction, such as holding village environmental protection public welfare competitions and villagers’ sports meetings. The aim is to achieve mutual communication between neighbors by organizing activities. (2) Taking full advantage of the internet. On the one hand, network media can publicize the hazards of domestic waste, separation knowledge as well as reward and punishment policies through multiple channels, so as to improve the environmental protection perception and policy cognition of farmers. On the other hand, it can improve the effectiveness of supervision and public opinion pressure, increase the opportunity cost of littering, which can promote the transformation of farmers’ willingness to separate domestic waste into behavior. (3) Implementation policies vary from person to person. In the future of domestic waste governance by local governments, policy formulation should take full account of the differences between different income groups and groups with political status, so as to improve the practicality and applicability of policies. In particular, it is important to activate the role of role models of high-income and politically elite farmers, thus enhancing the complementary effects between neighborhood effects and policy interventions. This is of great significance for improving the rural habitat and enhancing the well-being of the population.

There are some shortcomings in this study. First, this study uses cross-sectional data, which is not as reliable as panel data findings. Second, some control variables have been selected in the model of this paper, but there are inevitably individual omitted variables, leading to some bias in the results. Third, data limitations do not allow for a breakdown study of reward and punishment policies. Finally, due to the limitations of the survey area, the survey data in this paper can only reflect the situation in Jiangsu, and not reflect the whole country. This aspect can be attempted to be explored in future studies.

## Data availability statement

The raw data supporting the conclusions of this article will be made available by the authors, without undue reservation.

## Author contributions

XC: Methodology, Software, Writing – original draft, Writing – review & editing, Conceptualization. LX: Writing – original draft, Writing – review & editing, Methodology. BL: Methodology, Writing – original draft, Writing – review & editing. CW: Methodology, Writing – original draft, Writing – review & editing. YZ: Conceptualization, Methodology, Software, Writing – original draft, Writing – review & editing.
